# Synthesis and antibacterial activity of novel 5,6,7,8-tetrahydroimidazo[1,2-a]pyrimidine-2-carbohydrazide derivatives

**DOI:** 10.1186/s13065-015-0121-4

**Published:** 2015-09-24

**Authors:** Shashikala Kethireddy, Laxminarayana Eppakayala, Thirumala Chary Maringanti

**Affiliations:** Geethanjali College of Engineering and Technology, Keesara, Rangareddy, 501301 Telangana India; Mahatma Gandhi Institute of Technology, Gandipet, Hyderabad, 500 075 Telangana India; Jawaharlal Nehru Technological University Hyderabad, Kukatpally, Hyderabad, 500085 Telangana India

**Keywords:** Antibacterial activity, 2-Amino pyrimidine, Imidazo[1,2-a]pyrimidine-hydrazones, Norfloxacin

## Abstract

**Background:**

The intensely increasing multi-drug resistant microbial infections have encouraged the search for new antimicrobial agents. Hydrazone derivatives are known to exhibit a wide variety of biological activities including anti-microbial. In heterocyclic moiety, imidazo[1,2-a]pyrimidines are the subject of immense interest for their antimicrobial activity and also for their analgesic, antipyretic and anti-inflammatory properties.

**Results:**

Condensation of 5,6,7,8-tetrahydroimidazo[1,2-a]pyrimidine-2-carbohydrazide **7** with aromatic aldehydes **a–k** in ethanol at reflux led to the generation of hydrazone derivatives **8a–k** in 80–92% yield. The synthesis of carbohydrazide **7** was accomplished in six steps from commercially available 2-amino pyrimidine. The structures of the synthesized compounds were confirmed by ^1^H, ^13^C NMR, Mass and IR spectral data. All the synthesized hydrazone derivatives **8a–k** were tested in vitro for their antibacterial activity. Compounds **8d**, **8e** and **8f** exhibited excellent antibacterial activity with zone of inhibition 30–33 mm against *E. coli* (Gram negative bacteria) and *S. aureus* (Gram positive bacteria). These compounds also exhibited excellent antibacterial activity with zone of inhibition 22–25 mm against *P. aeruginosa* (Gram negative bacteria) and *S. pyogenes* (Gram positive bacteria).

**Conclusion:**

Synthesized and recorded antibacterial activity of some new 5,6,7,8-tetrahydro-imidazo[1,2-a]pyrimidine-hydrazone derivatives.

**Graphical abstract:**
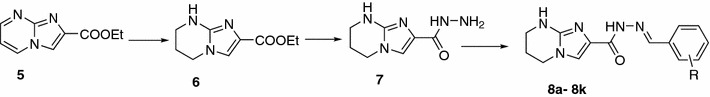
Synthesis of 5,6,7,8-tetrahydroimidazo[1,2-a]pyrimidine-2-carbohydrazide derivatives

## Background

The fast resistance of bacteria against antibiotics has become a prevailing medical problem. Treatment options for these infections are often inadequate especially in immune compromised patients. The intensely increasing multi-drug resistant microbial infections in the past few decades have become a serious health issue. The exploration of new antimicrobial agents will always remain as a major challenging task.

Hydrazones constitute a versatile compound of organic class having the basic structure R_1_R_2_C=NNH_2_. Hydrazones are formed by the replacement of the oxygen of carbonyl compounds with the –NNH_2_ functional group. The α-hydrogen atom of hydrazones is more acidic as compared to ketones [1, 2], because of its nucleophilic nature. Hydrazones are mainly synthesized by refluxing the appropriate quantity of substituted hydrazines/hydrazides with ketones and aldehydes in suitable solvents like ethanol, ethanol-glacial acetic acid, tetrahydrofuran, butanol, methanol, glacial acetic acid, etc. Hydrazone is a moiety that exhibits a wide variety of biological activities like anticonvulsant, antidepressant, analgesic, antimicrobial, antitumor, anti-platelet, vasodilator, antiviral, anticancer, antitubercular and anti-HIV [3–10].

Imidazo[1,2-a]pyrimidines are evolving as potentially interesting drugs particularly with regard to their antimicrobial, analgesic, antipyretic, anti-inflammatory properties and also with regard to their activity against ulcers [13]. They are also important as benzodiazepine receptor agonists, antiviral agents and calcium channel blockers [14].

Keeping in view of the biological importance of hydrazones and Imidazo[1,2-a]pyrimidines, in continuation to our research program about a potent antimicrobial agent, we report herein the synthesis, characterization and antibacterial activity of some new 5,6,7,8-tetrahydro-imidazo[1,2-a]pyrimidine-hydrazone derivatives. It is interesting to note that, a class of 5,6,7,8-tetrahydroimidazo[1,2-a]pyrimidine compounds is prescribed for treatment to reduce neurotoxic injury associated with anoxia or ischemia which typically follows stroke, cardiac arrest, hypoglycemia or perinatal asphyxia [11].

## Results and discussions

### Chemistry

**Scheme 1:**
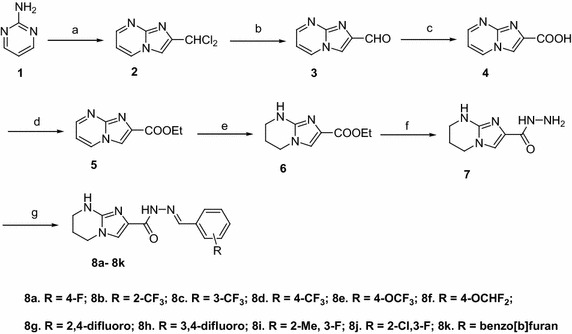
Synthesis of 5,6,7,8-tetrahydro-imidazo [1,2-a]pyrimidine-hydrazone derivatives **8a–k**. Reagents and conditions: *a* 1,1,3-trichloro acetone, NaHCO_3_, EtOH, reflux, 10 h; *b* CaCO_3_, water, reflux, 1 h; *c* oxone, DMF, 5°C, 2 h; *d* Conc; H_2_SO_4_, ethanol, reflux, 16 h; *e* 10% Pd–C, Ethanol, H_2_, 30 psi, 3 h; *f* hydrazine hydrate, Ethanol, reflux, 6 h; *g* benzaldehydes **a–k**, ethanol, reflux, 6 h.

The synthesis of 5,6,7,8-tetrahydro-imidazo[1,2-a]pyrimidine-hydrazone derivatives **8a–k** described in this paper were prepared according to the synthetic Scheme 1. The cyclo condensation reactions of 1,1,3-trichloro acetone with 2-aminopyrimidine **1** to 2-(dichloromethyl)imidazo[1,2-a]pyrimidine 2 was carried out in ethanol at reflux temperature for 10 h. Dichloromethyl imidazo[1,2-a]pyrimidine **2** was treated with CaCO_3_ in water at reflux for 1 h to produce imidazo[1,2-a]pyrimidine-2-carbaldehyde **3**. The oxidation of aldehyde **3** was carried out in presence of oxone to obtain the desired imidazo[1,2-a]pyrimidine-2-carboxylic acid **4**. Esterification of carboxylic acid **3** followed by the hydrogenation in presence of PtO_2_ at 30 psi resulted in the formation of ethyl 5,6,7,8-tetrahydroimidazo[1,2-a]pyrimidine-2-carboxylate **6**. Treatment of compound **6** with hydrazine-hydrate in ethanol at reflux temperature enabled in forming the key intermediate 5,6,7,8-tetrahydroimidazo[1,2-a]pyrimidine-2-carbohydrazide **7**. Condensation of carbohydrazide **7** with various aromatic aldehydes **a–k** in ethanol at reflux led to the generation of hydrazone derivatives **8a–k** in 80–92% yield (Scheme 1). The structures of the synthesized compounds were confirmed by ^1^H NMR, Mass and IR spectral data. As a representative example, the ^1^H NMR spectra of (E-*N*′-(4-(trifluoromethyl)benzylidene)-5,6,7,8-tetrahydroimidazo[1,2-a]pyrimidine-2-carbohydrazide **8d** is characterized as follows: the four singlets at 11.26, 8.52, 7.36 and 6.32 ppm with one proton integration corresponds to –CO–NH–N–, –N=HC–Ar, Imidazo-HC=C– and –CH_2_–NH– groups, respectively. The doublet signals at 7.85 and 7.78 ppm with two proton integration represents to 4-CF_3_ aromatic ring. The broad singlets with two proton integrations at 3.94, 3.35 and 1.95 ppm corresponds to the cyclic 5,6,7,8-tetrahydroimidazo ring. The mass spectra of the synthesized compounds showed (M+H) peaks, in agreement with their molecular formula. The IR data provided functional group evidence for the formation of the expected structures.

### Biological assay

The synthesized (E)-*N*′-(benzylidene)-5,6,7,8-tetrahydroimidazo[1,2-a]pyrimidine-2-carbohydrazide derivatives 8a–k were dissolved in dimethylsulphoxide at 250 μg/mL concentration (standard antibacterial drug, Norfloxacin was used as the reference antibiotic) and tested against Gram negative strains of (1) *Escherichia coli* (MTCC 443), (2) *Pseudomonas aeruginosa* (MTCC 424) and Gram positive strains of (3) *Staphylococcus aureus* (MTCC 96) and (4) *Streptococcus pyogenes* (MTCC 442) using agar well diffusion method according to the literature protocol [12, 13]. Activity was determined by zones showing complete inhibition (mm). Growth inhibition was calculated with reference to positive control. All the samples were taken in triplicates.

### Antibacterial activity

The screening results of antibacterial activity of hydrazone derivatives **8a–k** are summarized in Table 1. It is observed that compounds **8d**, **8e** and **8f** revealed excellent antibacterial activity with zone of inhibition 30–33 mm against *E. coli* (Gram negative bacteria) and *S. aureus* (Gram positive bacteria) even in the case of *P. aeruginosa* (Gram negative bacteria) and *S. pyogenes* (Gram positive bacteria), compounds 8d, 8e and 8f displayed excellent anti-bacterial activity with zone of inhibition 22–25 mm. Compounds **8a**, **8b** and **8c** showed appreciable activity while the compounds **8g** and **8h** showed moderate activity against all the tested bacterial strains. From the structural point of view of the scaffold, it may be generalized that scaffold with R = 4-CF_3_, 4-OCF_3_ and 4-OCHF_2_ showed excellent antibacterial activity, while R = 4-F, 2-CF_3_ and 3-CF_3_ showed good antibacterial activity and R = 2,4-difluoro and 3,4-difluoro showed moderate antibacterial activity. Furthermore, it is observed that within the series of the hydrazone derivatives 8a–k, compounds 8i (R = 2-Me, 3-F), 8j (R = 2-Cl, 3-F) and 8k (R = benzo[b]furan) showed no activity.

**Table 1:** Antibacterial activity of compounds **8a–k** (concentration used 250 μg/mL of DMSO)

Compound no.	Zones of inhibition of compounds 8a–k in mm			
	Gram negative		Gram positive	
	*E. coli* MTCC 443	*P. aeruginosa* MTCC 424	*S. aureus* MTCC 96	*S. pyogenes* MTCC 442
**8a**	25	19	24	17
**8b**	27	20	23	18
**8c**	26	19	23	17
**8d**	33	24	29	24
**8e**	30	25	30	22
**8f**	30	23	31	22
**8g**	20	16	19	13
**8h**	21	15	19	14
**8i**	–	–	–	–
**8j**	–	–	–	–
**8k**	–	–	–	–
Standard drug: Norfloxacin	29	23	28	21

### Experimental section

All commercial chemicals were taken as they are. The solvents underwent purification process as per standard procedures. For thin-layer chromatography (TLC) analysis, Merck pre-coated Plates (silica gel 60 F254) were used and spots were visualized with UV light. Merck silica gel 60 (230–400 mesh) was used for flash column chromatography and the eluting solvents were indicated in the procedures. Melting point (mp) determinations were performed by using Mel-temp apparatus and are uncorrected. ^1^H NMR spectra were recorded in Varian MR-400 MHz instrument. Chemical shifts were reported in δ parts per million (ppm) downfield from tetramethylsilane (TMS) with reference to internal standard and the signals were reported as s (singlet), d (doublet), dd (doublet of doublet), t (triplet), q (quartet), m (multiplet) and coupling constants in Hz. The mass spectra were recorded on Agilent ion trap MS. Infrared (IR) spectra were recorded on a Perkin Elmer FT-IR spectrometer. The benzaldehydes **a–k** utilized for the synthesis of **8a–k** were purchased from commercial sources.

### Synthesis of 2-(dichloromethyl)imidazo[1,2-a]pyrimidine 2

To a mixture of 2-aminopyrimidine (5 g, 52.57 mmol), sodium bicarbonate (11.34 g, 105.14 mmol) in ethylalcohol (20 mL) was added 1,1,3-trichloro acetone (12.70 g, 78.85 mmol) and stirred at reflux for 10 h. The completion of the reaction was monitored by TLC, the reaction mixture was poured into ice-water (30 mL) with vigorous stirring to precipitate the product, 2-(dichloromethyl)imidazo[1,2-a]pyrimidine **2**. The crude compound was taken to the next step without further purification.

### Synthesis of imidazo[1,2-a]pyrimidine-2-carbaldehyde 3

The compound **2** (1 g, 4.95 mmol) was dissolved in water (30 mL) and CaCO_3_ was added (5 g, 24.75 mmol) and refluxed for 1 h. The reaction mixture was cooled to room temperature and extracted with ethylacetate and evaporated under reduced pressure to obtain the crude compound **3**, which was purified by column chromatography (silica gel: 60–120 mesh, eluent: 1% MeOH/CHCl_3_) to afford compound **3**. Pale yellow solid, Yield: 60%; M.p: 209–210°C. ^1^H NMR (400 MHz, CDCl_3_): δ 9.96 (s, 1H), 9.66 (q, *J* = 6.8 Hz, 1H), 8.88 (q, *J* = 4.0 Hz, 1 H), 8.70 (s, 1H), 7.43 (q, *J* = 6.4 Hz, 1 H). ESI–MS: *m*/*z*, 148.2 (M+H)^+^.

### Synthesis of imidazo[1,2-a]pyrimidine-2-carboxylic acid 4

To a solution of compound **3** (2 g, 13.60 mmol) in DMF (15 vol), Oxone was added (40.80 mmol) at 5°C and stirred for 2 h. The completion of the reaction was monitored by TLC. As soon as the reaction was completed, the reaction mixture was diluted with water (45 mL) followed by ethyl acetate. The organic layer was washed with water followed by brine solution, dried over sodium sulphate, filtered and evaporated to produce compound **4** as pale yellow syrupy liquid. The crude compound was used in the next step without further purification. Yield: 1.10 g, 50%.

### Synthesis of ethyl imidazo[1,2-a] pyrimidine-2-carboxylate 5

To a solution of compound **4** (1.10 g, 6.75 mmol) in ethanol (12 mL), Conc. H_2_SO_4_ (catalyst) was added and refluxed for 16 h. The reaction mixture was evaporated and the residue was extracted with EtOAc (2 × 15 mL). The organic layer was washed with water followed by brine solution, dried over anhydrous sodium sulphate, filtered and evaporated to isolate compound **5** as a yellow viscous liquid. Yield: 1.06 g, 85%. M.p. 180–183°C; ^1^H NMR (400 MHz, CDCl_3_): δ 8.66 (dd, *J* = 2.1, 3.9 Hz, 1H), 9.50 (dd, *J* = 1.8, 6.9 Hz, 1H), 8.15 (s, 1H), 6.96 (dd, *J* = 4.2, 6.9 Hz, 1H), 4.45 (q, *J* = 7.2 Hz, 2H), 1.44 (t, *J* = 7.2 Hz, 3H). ESI–MS: *m*/*z*, 192.0 (M+H)^+^

### Synthesis of ethyl 5,6,7,8-tetrahydroimidazo[1,2-a]pyrimidine-2-carboxylate 6

A solution of compound **5** (1.06 g, 5.55 mmol) in ethanol (20 mL): conc. HCl (0.5 mL) was hydrogenated at 30 psi in the presence of 20% PtO_2_ (200 mg) for 3 h. The reaction mixture was filtered under nitrogen atmosphere and the filtrate was evaporated to obtain compound **6**. The pH of the solution was adjusted to 9–10 with aqueous sodium carbonate solution and extracted with chloroform (2 × 25 mL). The combined organic extracts were washed with water (50 mL) followed by brine solution. The organic layer was dried over anhydrous sodium sulphate and concentrated in vacuum to isolate compound **6** as yellow solid. Yield: 0.97 g, 90%; M.p: 170–171°C; ^1^H NMR (400 MHz, CDCl_3_): δ 7.18 (s, 1H), 6.25 (brs, 1H), 1.36 (t, *J* = 7.2 Hz, 3H), 2.07 (t, *J* = 5.7 Hz, 2H), 3.45 (t, *J* = 5.4 Hz, 2H), 3.90 (t, *J* = 6.0 Hz, 2H), 4.32 (q, *J* = 7.2 Hz, 2H), ESI–MS: *m*/*z*, 196.2 (M+H)^+^.

### Synthesis of 5,6,7,8-tetrahydroimidazo[1,2-a]pyrimidine-2-carbohydrazide 7

A mixture of compound **6** (0.95 g, 4.87 mmol) and hydrazine hydrate (5.0 mmol) in ethanol (4 mL) was refluxed for 6 h. The reaction mixture was evaporated and the residue was diluted with EtOAc and water. The organic layer was washed with water followed by brine solution, separated, dried over anhydrous Na_2_SO_4_, filtered and evaporated to obtain compound **7** as a pale yellow solid. Yield: 80%; M.p: 116–117°C; ^1^H NMR (400 MHz, CDCl_3_): δ 8.50 (s, 1H), 7.09 (s, 1H), 6.34 (s, 1H), 4.20 (brs, 1H), 3.86 (t, *J* = 6.0 Hz, 2H), 3.21–3.17 (m, 2H), 1.93–1.87 (m, 2H); ESI–MS: *m*/*z*, 181.8 (M+H)^+^.

### General experimental procedure for the synthesis of hydrazone derivatives (8a–k)

Respective benzaldehydes **a–k** (1.0 mmol) were added to an ethanol solution containing **7**(100 mg, 0.553 mmol) and the contents were stirred at reflux temperature for 6 h. Ethanol was evaporated from the reaction mixture and the residue was extracted with pet-ether, to obtain pure compounds. Yields of the products varied between 80 and 92%.

#### (E)-*N*′-(4-fluorobenzylidene)-5,6,7,8-tetrahydroimidazo[1,2-a]pyrimidine-2-carbohydrazide (8a)

M.p: 84–85°C; IR(KBr): υ_max_ 3,443, 3,303, 3,275, 3,243, 3,187, 2,981, 1,667, 1,612, 1,602, 1,565, 1,506, 1,495, 1,425, 1,415, 1,370, 1,350, 1,319, 1,290, 1,275, 1,236, 1,209, 1,192, 1,183, 1,156, 1,135, 1,112, 1,101, 1,064, 998, 959, 930, 901, 854, 837, 819, 788, 751, 629, 612, 566, 519, 505 cm^−1^; ^1^H NMR (400 MHz, DMSO-d_6_): δ 11.01 (s, 1H), 8.43 (s, 1H), 7.71–7.68 (m, 2H), 7.32–7.25 (m, 3H), 6.30 (s, 1H), 3.39 (t, *J* = 5.6 Hz, 2H), 3.24 (brm, 2H), 1.94 (t, *J* = 5.6 Hz, 2H); ESI–MS: *m*/*z*, 287.9 (M+H)^+^.

#### (E)-*N*′-(2-(trifluoromethyl) benzylidene)-5,6,7,8-tetrahydroimidazo[1,2-a]pyrimidine-2-carbohydrazide (8b)

M.p: 104–105°C; ^1^H NMR (400 MHz, DMSO-d_6_): δ 11.22 (s, 1H), 8.86 (s, 1H), 8.16 (d, *J* = 8.4 Hz, 1H), 7.76 (d, *J* = 8.4 Hz, 1H), 7.74–7.59 (m, 2H), 7.35 (s, 1H), 6.28 (s, 1H), 3.93 (brs, 2H), 3.24 (brs, 2H), 1.94 (brs, 2H); ESI–MS: *m*/*z*, 337.9 (M+H)^+^.

#### (E)-*N*’-(3-(trifluoromethyl)benzylidene)-5,6,7,8-tetrahydroimidazo[1,2-a]pyrimidine-2-carbohydrazide (8c)

M.p: 98–99°C; ^1^H NMR (400 MHz, DMSO-d_6_): δ 11.22 (s, 1H), 8.86 (s, 1H), 8.16 (d, *J* = 8.4 Hz, 1H), 7.76 (d, *J* = 8.4 Hz, 1H), 7.74–7.59 (m, 2H), 7.35 (s, 1H), 6.28 (s, 1H), 3.93 (brs, 2H), 3.24 (brs, 2H), 1.94 (brs, 2H); ESI–MS: *m*/*z*, 337.9 (M+H)^+^.

#### (E)-*N*’-(4-(trifluoromethyl) benzylidene)-5,6,7,8-tetrahydroimidazo[1,2a]pyrimidine-2-carbohydrazide (8d)

M.p: 120–121°C; IR(KBr): υ_max_ 3,443, 3,261, 3,242, 3,184, 3,147, 3,131, 3,085, 3,011, 2,982, 2,959, 2,936, 2,851, 2,206, 1,663, 1,625, 1,560, 1,522, 1,507, 1,494, 1,463, 1,445, 1,433, 1,413, 1,364, 1,331, 1,321, 1,300, 1,293, 1,271, 1,235, 1,210, 1,193, 1,182, 1,158, 1,134, 1,119, 1,102, 1,068, 1,017, 994, 967, 959, 939, 898, 895, 842, 832, 784, 762, 720, 709, 672, 644, 630, 615, 594, 508, 503 cm^−1 1^H NMR (400 MHz, DMSO-d_6_): δ 11.26 (s, 1H), 8.52 (s, 1H), 7.85 (d, *J* = 8.4 Hz, 2H), 7.78 (d, *J* = 8.0 Hz, 2H), 7.36 (s, 1H), 6.32 (s, 1H), 3.94 (brs, 2H), 3.35 (brs, 2H), 1.95 (brs, 2H); ESI–MS: *m*/*z*, 337.8 (M+H)^+^.

#### (E)-*N*′-(4-(trifluoromethoxy)benzylidene)-5,6,7,8-tetrahydroimidazo[1,2-a]pyrimidine-2-carbohydrazide (8e)

M.p: 126–127°C; IR(KBr): υ_max_ 3,243, 3,224, 3,182, 3,099, 3,054, 2,972, 2,883, 2,847, 1,653, 1,631, 1,605, 1,555, 1,535, 1,506, 1,492, 1,441, 1,414, 1,386, 1,367, 1,351, 1,325, 1,301, 1,270, 1,243, 1,217, 1,193, 1,166, 1,099, 1,090, 1,072, 1,017, 1,002, 964, 944, 918, 902, 878, 850, 793, 756, 715, 677, 650, 662, 600, 532 cm^−1^; ^1^H NMR (400 MHz, DMSO-d_6_): δ 11.10 (s, 1H), 8.46 (s, 1H), 7.76 (d, *J* = 8.4 Hz, 2H), 7.42 (d, *J* = 8.4 Hz, 2H), 7.33 (s, 1H), 6.30 (s, 1H), 3.92 (t, *J* = 6.0 Hz, 2H), 3.24 (brs, 2H), 1.94 (brs, 2H); ESI–MS: *m*/*z*, 354.0 (M+H)^+^.

#### (E)-*N*′-(4-(difluoromethoxy)benzylidene)-5,6,7,8-tetrahydroimidazo[1,2-a]pyrimidine-2-carbohydrazide (8f)

M.p: 119–120°C; IR(KBr): υ_max_ 3,429, 3,272, 3,099, 3,056, 3,041, 2,972, 2,920, 2,886, 2,847, 1,644, 1,603, 1,575, 1,538, 1,508, 1,456, 1,440, 1,417, 1,372, 1,323, 1,295, 1,278, 1,231, 1,214, 1,203, 1,189, 1,174, 1,124, 1,115, 1,100, 1,023, 961, 936, 903, 864, 836, 789, 717, 702, 679, 647, 617, 582, 561, 529, 492, 479, 459 cm^−1^; ^1^H NMR (400 MHz, DMSO-d_6_): δ 11.02 (s, 1H), 8.43 (s, 1H), 7.70 (d, *J* = 8.8 Hz, 2H), 7.31 (s, 1H), 7.22 (d, *J* = 8.4 Hz, 2H), 6.30 (s, 1H), 3.92 (t, *J* = 5.6 Hz, 2H), 3.24 (brs, 2H), 1.94 (t, J = 5.8 Hz, 2H); ESI–MS: *m*/*z*, 336.0 (M+H)^+^.

#### (E)-*N*′-(2,4-difluorobenzylidene)-5,6,7,8-tetrahydroimidazo[1,2-a]pyrimidine-2-carbohydrazide (8 g)

M.p: 138–139°C; IR(KBr): υ_max_ 3,429, 3,265, 3,193, 3,139, 3,120, 3,097, 3,008, 2,981, 2,962, 2,882, 2,562, 1,665, 1,617, 1,561, 1,517, 1,492, 1,459, 1,427, 1,372, 1,316, 1,282, 1,214, 1,190, 1,175, 1,140, 1,097, 1,066, 995, 945, 897, 854, 828, 820, 782, 744, 728, 718, 707, 638, 629, 610, 577, 542, 509; cm^−1^; ^1^HNMR (400 MHz, DMSO-d_6_): δ 11.32 (s, 1H), 8.64 (s, 1H), 7.96–7.90 (m, 1H), 7.35 (d, *J* = 2.8 Hz, 1H), 7.18 (t, *J* = 8.8 Hz, 1H), 6.27 (s, 1H), 3.92 (t, *J* = 6.0 Hz, 2H), 3.24 (brs, 2H), 1.94 (t, *J* = 5.2 Hz, 2H); ESI–MS: *m*/*z*, 305.0 (M+H)^+^.

#### (E)-*N*′-(3,4-difluorobenzylidene)-5,6,7,8-tetrahydroimidazo[1,2-a]pyrimidine-2-carbohydrazide (8h)

M.p: 143–144°C; IR(KBr): υ_max_ 3,415, 3,307, 3,249, 3,114, 2,884, 2,559, 1,672, 1,617, 1,513, 1,437, 1,423, 1,370, 1,301, 1,283, 1,231, 1,205, 1,137, 1,110, 1,083, 988, 937, 918, 889, 876, 796, 767, 716, 636, 578, 537, 515 cm^−1^; ^1^H NMR (400 MHz, DMSO-d_6_): δ 11.30 (s, 1H), 8.48 (s, 1H), 7.88 (s, 1H), 7.68-7.19 (m, 3H), 6.30 (s, 1H), 3.93 (brs, 2H), 3.40 (brs, 2H), 1.95 (brs, 2H); ESI–MS: *m*/*z*, 306.0 (M+H)^+^.

#### (E)-*N*′-(4-fluoro-2-methylbenzylidene)-5,6,7,8-tetrahydroimidazo[1,2-a]pyrimidine-2-carbohydrazide (8i)

M.p: 81–82°C; IR(KBr): υ_max_ 3,419, 3,170, 3,097, 3,060, 2,967, 2,906, 2,835, 1,650, 1,633, 1,594, 1,556, 1,492, 1,456, 1,442, 1,417, 1,391, 1,379, 1,368, 1,352, 1,324, 1,300, 1,266, 1,238, 1,225, 1,194, 1,152, 1,099, 1,067, 1,004, 992, 962, 955, 896, 826, 811, 717, 698, 617, 577, 517 cm^−1^; ^1^H NMR (400 MHz, DMSO-d_6_): δ 11.00 (s, 1H), 8.72 (s, 1H), 7.86 (d, *J* = 8.4 Hz, 1H), 7.30 (s, 1H), 7.10-7.05 (m, 2H), 6.28 (s, 1H), 3.92 (t, *J* = 5.6 Hz, 2H), 3.24 (brs, 2H), 1.94 (brs, 2H); ESI–MS: *m*/*z*, 302.10 (M+H)^+^.

#### (E)-*N*′-(2-chloro-3-(trifluoromethyl)benzylidene)-5,6,7,8-tetrahydroimidazo[1,2-a]pyrimidine-2-carbohydrazide (8j)

M.p: 117–119°C; ^1^H NMR (400 MHz, DMSO-d_6_): δ 11.58 (s, 1H), 8.88 (s, 1H), 8.20 (d, *J* = 8.4 Hz, 1H), 7.88 (d, *J* = 8.6 Hz, 1H), 7.58 (t, *J* = 7.8 Hz, 1H), 7.30 (s, 1H), 6.25 (s, 1H), 3.94 (t, *J* = 5.8 Hz, 2H), 3.22 (brs, 2H), 1.94 (t, *J* = 5.4 Hz, 2H); ESI–MS: *m*/*z*, 372.13 (M+H)^+^.

#### (E)-*N*′-((benzofuran-2-yl)methylene)-5,6,7,8-tetrahydroimidazo[1,2-a]pyrimidine-2-carbohydrazide (8k)

M.p: 117–119°C; IR(KBr): υ_max_ 3,366, 3,239, 3,135, 2,964, 2,930, 2,881, 1,659, 1,607, 1,574, 1,560, 1,506, 1,492, 1,474, 1,448, 1,406, 1,366, 1,331, 1,317, 1,290, 1,242, 1,219, 1,196, 1,158, 1,144, 1,131, 1,106, 1,095, 1,073, 1,004, 992, 952, 943, 891, 873, 839, 824, 764, 715, 659, 613, 580, 544, 468, 459, 440 cm^−1^; ^1^H NMR (400 MHz, DMSO-d_6_): δ 11.30 (s, 1H), 8.50 (s, 1H), 7.60–7.48 (m, 2H), 7.32–7.28 (m, 1H), 7.18 (s, 1H), 6.30 (s, 1H), 3.94 (t, *J* = 5.8 Hz, 2H), 3.40 (brs, 2H), 1.80 (brs, 2H); ESI–MS: *m*/*z*, 310.14 (M+H)^+^.

## Conclusion

The synthesis of 5,6,7,8-tetrahydro-imidazo[1,2-a]pyrimidine-hydrazone derivatives **8a–k** described in this paper was prepared in six elaborate steps from commercially available 2-aminopyrimidine as starting material. All the synthesised compounds 8a–k were investigated for their in vitro antimicrobial activity. Compounds **8d**, **8e** and **8f** demonstrated excellent antibacterial activity with zone of inhibition 30–33 mm against *E. coli* (Gram negative bacteria) and *S. aureus* (Gram positive bacteria) These compounds also displayed excellent antibacterial activity with zone of inhibition 22–25 mm even in the case of *P. aeruginosa*(Gram negative bacteria) and *S. pyogenes*(Gram positive bacteria).
